# The study of trypanosome species circulating in domestic animals in two human African trypanosomiasis foci of Côte d'Ivoire identifies pigs and cattle as potential reservoirs of *Trypanosoma brucei gambiense*

**DOI:** 10.1371/journal.pntd.0005993

**Published:** 2017-10-18

**Authors:** Martial Kassi N’Djetchi, Hamidou Ilboudo, Mathurin Koffi, Jacques Kaboré, Justin Windingoudi Kaboré, Dramane Kaba, Fabrice Courtin, Bamoro Coulibaly, Pierre Fauret, Lingué Kouakou, Sophie Ravel, Stijn Deborggraeve, Philippe Solano, Thierry De Meeûs, Bruno Bucheton, Vincent Jamonneau

**Affiliations:** 1 Laboratoire des Interactions Hôte-Microorganisme-Environnement et Evolution, Unité de Formation et de Recherche Environnement, Université Jean Lorougnon Guédé, Daloa, Côte d’Ivoire; 2 Unité de recherches sur les bases biologiques de la lutte intégrée, Centre International de Recherche-Développement sur l’Elevage en zone Subhumide, Bobo-Dioulasso, Burkina Faso; 3 Unité de Formation et de Recherche Sciences et Techniques, Université Nazi Boni, Bobo-Dioulasso, Burkina-Faso; 4 Unité de Recherche « Trypanosomoses », Institut Pierre Richet, Bouaké, Côte d’Ivoire; 5 Unité Mixte de Recherche IRD-CIRAD 177, INTERTRYP, Institut de Recherche pour le Développement (IRD), Montpellier, France; 6 Programme National d’Elimination de la Trypanosomose Humaine Africaine, Ministère de la Santé et de l’Hygiène Publique, Abidjan, Côte d’Ivoire; 7 Biomedical Sciences Department, Institute of Tropical Medicine, Antwerp, Belgium; Institute of Tropical Medicine, BELGIUM

## Abstract

**Background:**

Important control efforts have led to a significant reduction of the prevalence of human African trypanosomiasis (HAT) in Côte d’Ivoire, but the disease is still present in several foci. The existence of an animal reservoir of *Trypanosoma brucei gambiense* may explain disease persistence in these foci where animal breeding is an important source of income but where the prevalence of animal African trypanosomiasis (AAT) is unknown. The aim of this study was to identify the trypanosome species circulating in domestic animals in both Bonon and Sinfra HAT endemic foci.

**Methodology/Principal findings:**

552 domestic animals (goats, pigs, cattle and sheep) were included. Blood samples were tested for trypanosomes by microscopic observation, species-specific PCR for *T*. *brucei* sl, *T*. *congolense*, *T*. *vivax* and subspecies-specific PCR for *T*. *b*. *gambiense* and *T*. *b*. *gambiense* immune trypanolysis (TL). Infection rates varied significantly between animal species and were by far the highest in pigs (30%). *T*. *brucei* s.l was the most prevalent trypanosome species (13.7%) followed by *T*. *congolense*. No *T*. *b*. *gambiense* was identified by PCR while high TL positivity rates were observed using *T*. *b*. *gambiense* specific variants (up to 27.6% for pigs in the Bonon focus).

**Conclusion:**

This study shows that domestic animals are highly infected by trypanosomes in the studied foci. This was particularly true for pigs, possibly due to a higher exposure of these animals to tsetse flies. Whereas *T*. *brucei* s.l. was the most prevalent species, discordant results were obtained between PCR and TL regarding *T*. *b*. *gambiense* identification. It is therefore crucial to develop better tools to study the epidemiological role of potential animal reservoir for *T*. *b*. *gambiense*. Our study illustrates the importance of “one health” approaches to reach HAT elimination and contribute to AAT control in the studied foci.

## Author summary

In Africa, significant efforts to control human African trypanosomiasis (HAT) over the past three decades have drastically reduced the prevalence of the disease and elimination seems today an achievable goal. However, potential animal reservoirs of *Trypanosoma brucei gambiense* may compromise this ambitious objective. In the Bonon and Sinfra HAT endemic foci in Côte d’Ivoire, no recent data are available about the prevalence of animal African trypanosomiasis (AAT). The aim of this study was to identify trypanosomes circulating in domestic animals in these two HAT foci using serological, parasitological and molecular tools. We showed that *T*. *brucei* s.l. and *T*. *congolense* were the most prevalent trypanosome species and that pigs and cattle were the most infected animals. Discordant results were observed between the *T*. *b*. *gambiense* specific molecular and serological tools and the presence of an animal reservoir for *T*. *b*. *gambiense* remains unclear. Nevertheless, improved control strategies can be proposed based on this study to reach HAT elimination and contribute to AAT control in the study areas.

## Introduction

Human African trypanosomiasis (HAT) or sleeping sickness is a vector borne parasitic disease caused by *Trypanosoma brucei gambiense* (*T*. *b*. *gambiense*) in West and Central Africa and *T*. *b*. *rhodesiense* in East Africa. *T*. *b*. *gambiense* is responsible for 98% of all HAT cases reported in the last decade and remains an important public health concern in sub-Saharan Africa [1]. However, with less than 3000 cases reported in 2015 [2], HAT elimination seems an achievable goal [3]. A similar situation occurred in the 1960s but, after an early sense of victory, the disease reemerged. *Gambiense* HAT is generally considered as an anthroponotic disease, but the absence of animal reservoirs has never been demonstrated. The existence of an animal reservoir for *T*. *b*. *gambiense* could be one of the factors that causes reemergence of the disease after successful control campaigns [4].

In Côte d’Ivoire, significant efforts to control the disease over the past three decades have been made and drastically reduced the prevalence of HAT [5]. The last epidemic was contained in the Sinfra and Bonon foci at the early2000s [6–8]. Despite continuous control efforts, few HAT cases are still passively diagnosed from these two foci as well as from historical foci of the Western-Centre part of the country [7–9]. Transmission persistence may be due to the existence of a residual chronic human and/or animal reservoir of *T*. *b*. *gambiense* in these areas where tsetse flies are still present [10–13].

Several studies have highlighted the importance of wild and domestic animals in the transmission cycle of *T*. *b*. *rhodesiense* [14,15], but this is still under debate for *T*. *b*. *gambiense*. Noteworthy, the presence of *T*. *b*. *gambiense* in domestic and wild animals have been reported in Cameroun [16] and Equatorial Guinea [17–19]. In Côte d’Ivoire, such studies are scarce and the last report dates from the early 2000s in which the authors investigated the presence of trypanosomes in pigs in the Bonon HAT focus. High prevalence of *T*. *brucei* s.l. was observed but the presence of *T*. *b*. *gambiense* in this animal species remained unclear [20]. It is crucial to increase the efforts in studying the existence of an animal reservoir of *T*. *b*. *gambiense* as this could compromise HAT elimination.

No data are available regarding the prevalence of *T*. *b*. *brucei*, *T*. *congolense* and *T*. *vivax* animal African trypanosomiasis (AAT), in the Bonon and Sinfra foci, despite that animal breeding represents an increasing source of food and income in these areas with high human population densities.

In the context of the one health approach that was recently suggested for HAT and AAT control [21], the aim of the present study was to characterize trypanosomes circulating in domestic animals in the HAT foci of Bonon and Sinfra in Côte d’Ivoire. We used *Trypanosoma* species-specific PCR assays, microsatellite genotyping and immune trypanolysis (TL) with three variant antigenic types (VAT) of which two are specific for *T*. *b*. *gambiense* [22]. We show that *T*. *brucei* s.l. and *T*. *congolense* were the most prevalent trypanosome species in the two foci and that pigs and cattle were the most infected animals, with *T*. *brucei* s.l. and *T*. *congolense* respectively. Discordant results were observed between the *T*. *b*. *gambiense* specific PCR and TL tests and the existence of an animal reservoir of *T*. *b*. *gambiense* thus remains unclear.

## Materials and methods

### Study area

**Fig 1:**
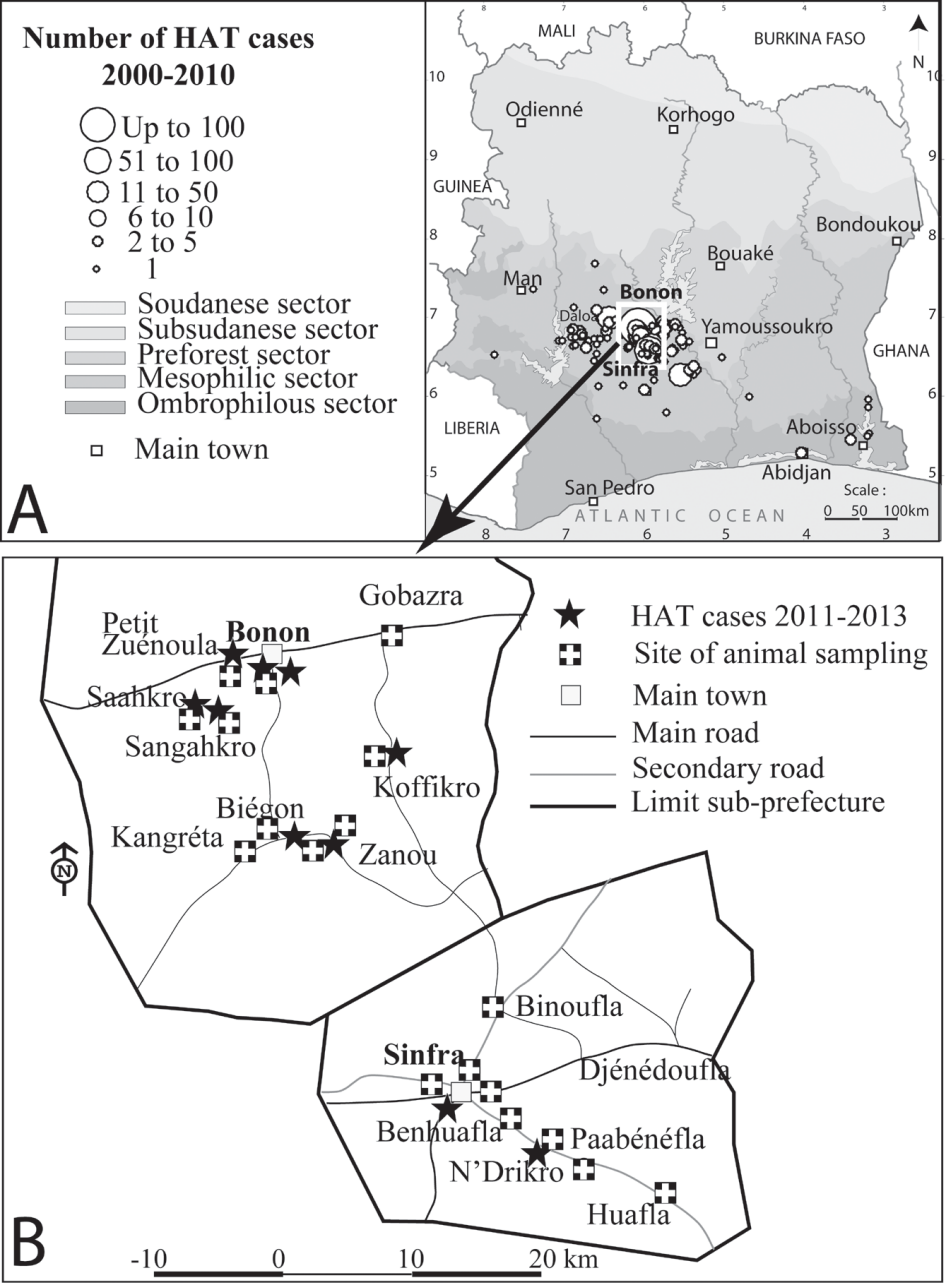
The study areas and sites of animal sampling. A. Localization of the Bonon and Sinfra foci which reported the highest number of HAT cases diagnosed from 2000 to 2010 in Côte d’Ivoire. B. Localization of the last HAT cases diagnosed from 2011 to 2013 and the sites of domestic animals sampling in the Bonon and Sinfra foci. This figure was created by the mapping service of our team based at Institut Pierre Richet (Bouaké, Côte d’Ivoire) specifically for this manuscript.

The study was carried out in September/October 2013 in the Sinfra and Bonon areas, which are located in the western-central part of Côte d’Ivoire (Fig 1). In recent decades, the mesophyle forest has been progressively replaced by cash crops (mainly cocoa and coffee, but also bananas, cassava, rice and yam) leading to a favorable environmental context for HAT development in these areas [23]. The evolution of HAT prevalence in Côte d’Ivoire is well documented since the 1950s. The number of cases diagnosed from 2000 to 2010 (Fig 1A) shows that these two foci were the most endemic during this period. Control efforts conducted from 1995 till present could largely contain the epidemic [7,11] but few cases are still passively diagnosed each year [7]. Based on the last 10 HAT cases who were diagnosed from 2011 to 2013, we identified 8 and 10 study sites in the Sinfra and Bonon foci, respectively (Fig 1B). These 18 study sites (less than 10 km from the last detected HAT cases) are expected to be those where transmission is still active and where we had the highest chance to detect *T*. *b*. *gambiense* in domestic animals.

We focused our study on cattle (Zebu), goats, sheep and pigs since they are the most common domestic animals in the study areas. They are mainly bred in the periphery of villages or along the small rivers crossing the villages, where tsetse flies are often abundant [10,12,24–26]. Generally, sheep, goats and cattle freely graze during the day and are kept in enclosures at night while pigs freely roam day and night.

### Sample collection and parasitological investigation in the field

For each animal, 5ml of blood was taken from the jugular vein. Parasitological diagnosis was performed in the field by microscopic examination using the buffy coat technique (BCT) [27]. BCT was considered positive when trypanosomes could be visually detected regardless of the species. In addition, 1 ml of plasma and 1 ml of blood were aliquoted and immediately frozen at -20°C during transport and subsequently at -80°C in the lab for PCR and immune trypanolysis testing.

### PCR analysis

DNA from 500 μL of blood was extracted using the DNeasy Tissue kit (Qiagen, Valencia, CA, USA) as described previously [28] and subjected to diagnostic PCR assays using *Trypanosoma* species specific primers for *T*. *brucei* s.l. (TBR1-2) [29], *T*. *congolense* forest type (TCF1-2) [30], *T*. *congolense* savannah type (TCS1-2) [31], *T*. *vivax* (TVW1-2) [30]. Positives samples for *T*. *brucei* s.l were tested for *T*. *b*. *gambiense* using primers targeting the TgsGP gene [32]. All PCR reactions were carried out using 5 μl of DNA template in a reaction volume of 50 μL 1xPCR reaction buffer comprising 0.2 mM of dNTP, 0.2 μM of each primer and 2.5 U of Taq polymerase. A positive control was added to the corresponding PCR and distilled water was used as negative control. The PCR products were visualized by electrophoresis in a 2% agarose gel stained with GelRed and illuminated with UV light.

### Microsatellite genotyping

Samples positive in the *T*. *brucei* s.l. specific TBR PCR were further characterized by seven microsatellite markers Ch1/18, Ch1/D2/7 [33], M6C8 [34], Micbg5, Micbg6, Misatg4, Misatg9 [35], as previously described [36]. Reference stocks of *T*. *b*. *gambiense* (n = 18), *T*. *b*. *gambiense* group 2 (n = 3), *T*. *b*. *brucei* (n = 1) and *T*. *b*. *rhodesiense* (n = 1) were included. A neighbor-joining tree was computed under multiple sequence alignment (MSA) [37] with Mega 5 [38] on a Cavalli-Sforza and Edward's chord distance matrix [39] as recommended by Takezaki et al. [40].

### Trypanolysis test

Plasma samples were analyzed with the immune trypanolysis test (TL) using cloned populations of *T*. *b*. *gambiense* variant antigen type (VATs) LiTat 1.3, LiTat 1.5 and LiTat 1.6 as previously described [22,41]. LiTat 1.3 and LiTat 1.5 VATs are reported to be specific for *T*. *b*. *gambiense*, while LiTat 1.6 VAT can both be expressed in *T*. *b*. *gambiense* and *T*. *b*. *brucei* [22].

### Statistical analysis

All statistical analyses were done with JMP11 (SAS Institute). Proportions of positive animals for BCT, PCR and TL were compared regarding foci and host species using the Chi-square analysis.

### Ethics statement

Sample collection was conducted within the framework of epidemiological surveillance activities supervised by the HAT National Elimination Program (HAT NEP). No ethical statement is required by local authorities. Any veterinarian may carry out blood sampling on domestic animals, with the authorization of the owner, as it is performed during prophylaxis or diagnostic campaign. No samples other than those for routine screening and diagnostic procedures were collected. Breeders gave their consent for animal sampling after explaining the objectives of the study. For animal care, venous sampling was performed by a veterinary of the Laboratoire National d’Appui au Développement Rural (Ministry of Agriculture). A deworming treatment (Bolumisol, Laprovet) was provided free to all animals sampled and those positive with BCT were treated for trypanosomiasis.

## Results

In total, 552 animals were sampled of which 251 from Bonon and 301 from Sinfra as presented in Table 1.

**Table 1:** Number of domestic animals sampled by species and foci.

	Bonon	Sinfra	Total
Cattle	35	52	87
Goats	47	89	136
Sheep	92	100	192
Pigs	77	60	137
Total	251	301	552

### Parasitology

**Fig 2:**
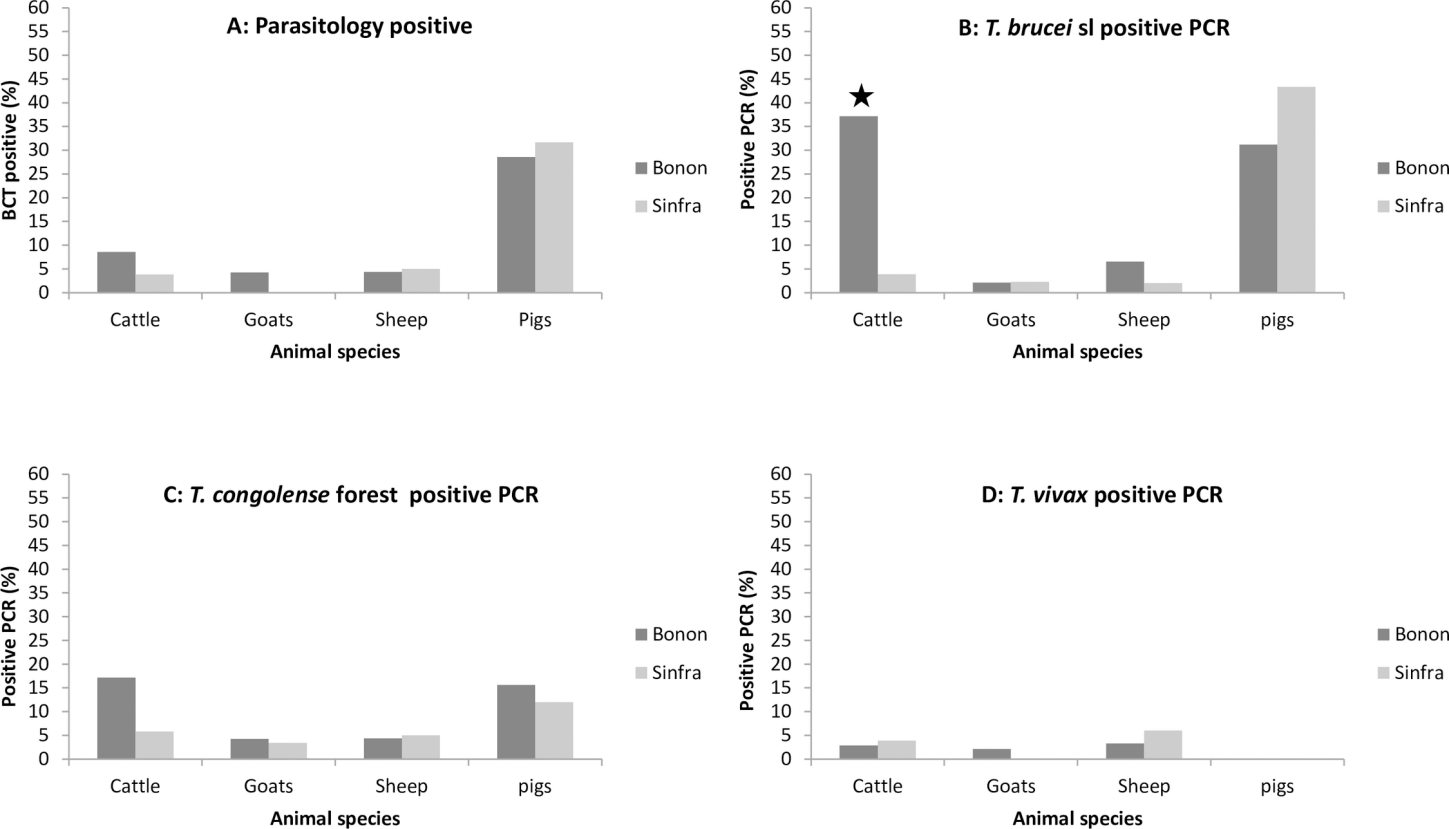
Parasitological and PCR results. Proportion of BCT (2A), *T*. *brucei* s.l. TBR-PCR (2B), *T*. *congolense* forest type TCF-PCR (2C) and *T*. *vivax* TVW-PCR (2D) positive results on the total sample collection for each host in the two foci. A significant difference between Bonon and Sinfra is indicated by a star.

Out of the 552 animals sampled, 57 trypanosome infections (10.3%) were detected by BCT (Table 2). Highest prevalence was observed in pigs (*p*<0.0001) with a prevalence of almost 30%. No significant differences were observed between the two foci in the infection rates (Fig 2A).

**Table 2:** BCT and PCR results per host species (percentage are given in brackets).

Host	BCT	TBR+TCF+TVW PCR	TBR PCR	TCF PCR	TVW PCR
Cattle	5 (5.75)	22 (25.28)	15 (17.24)	9 (10.34)	3 (3.45)
Goats	2 (1.47)	9 (6.61)	3 (2.21)	5 (3.68)	1 (0.73)
Sheep	9 (4.69)	21 (10.93)	8 (4.17)	9 (4.69)	9 (4.69)
Pigs	41 (29.93)	57 (41.6)	50 (36.50)	24 (17.52)	0 (0)
Total	57 (10.33)	109 (19.74)	76 (13.77)	47 (8.51)	13 (2.35)

### Molecular diagnosis

No animals were positive with the TCS specific primers. In total, 109 trypanosome infections (19.7%) were detected with at least one PCR (TBR, TCF or TVW) with the highest prevalence observed in pigs (41.6%, Table 2). *T*. *bucei* s.l. was the most prevalent trypanosome species (13.8%) followed by *T*. *congolense* forest type (8.5%) and *T*. *vivax* (2.4%). No *T*. *vivax* infection was detected in pigs. Mixed infections with positive results in at least two different PCR assays were observed in 29 animals. Most were mixed infections with *T*. *bucei* s.l. / *T*. *congolense* forest type and mainly observed in pigs (59%).

TBR, TCF and TVW PCR results for the different hosts in both foci are presented in Fig 2B, 2C and 2D. Pigs and cattle showed higher TBR and TCF-PCR positivity rates compared to sheep and goats. No significant differences were observed between the two foci except for TBR PCR positivity rates in cattle. The highest PCR positivity rate was observed in pigs in Sinfra (43.3%). All 76 DNA samples positive in the TBR PCR were further analyzed with the *T*. *b*. *gambiense* specific TgsGP PCR and all were negative.

### Microsatellite genotyping

**Fig 3:**
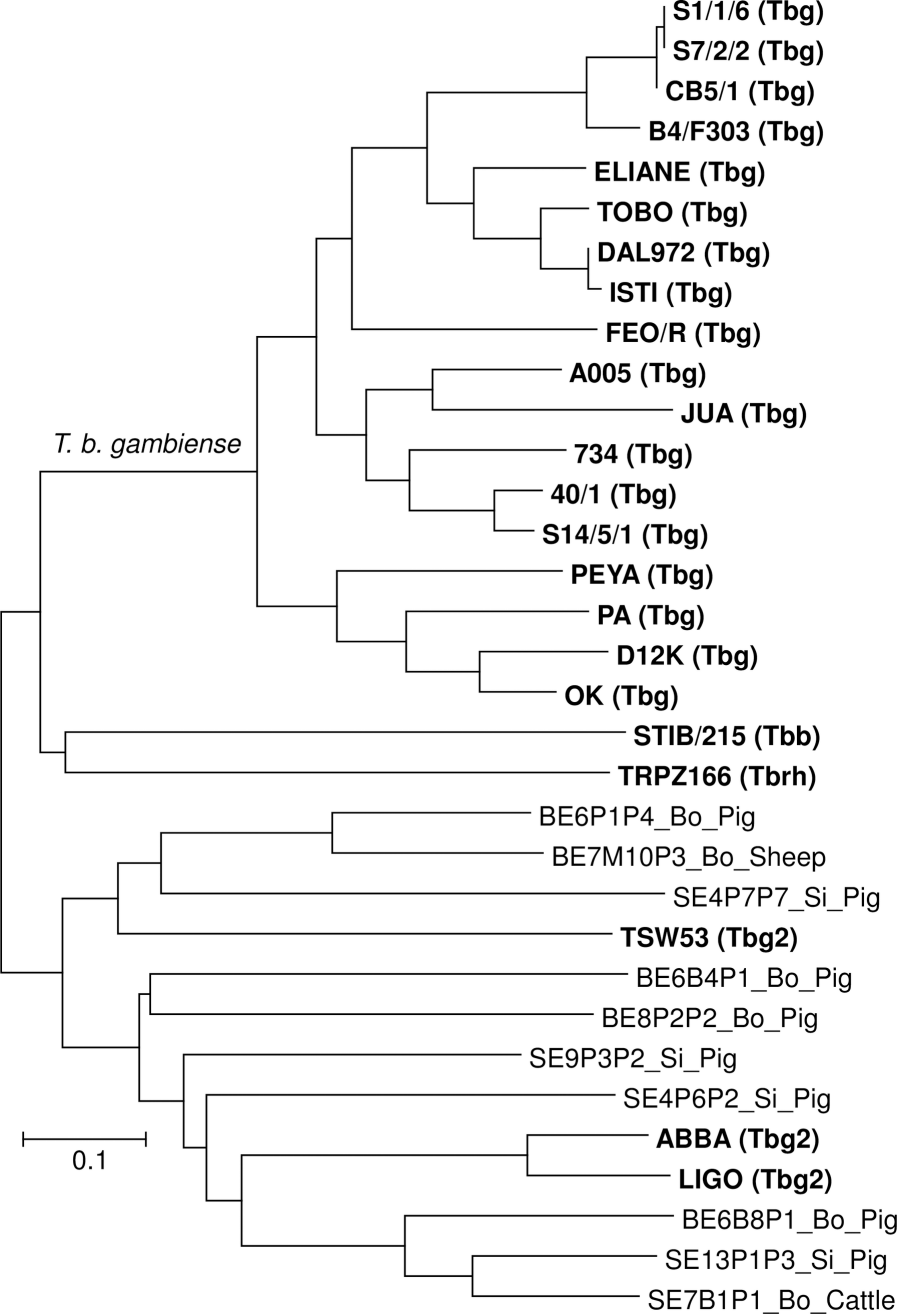
Microsatellite genotyping results. Neighbor-joining tree (NJTree), based on Cavalli-Sforza and Ewards Chord distance, of the amplified microsatellite genotypes. Reference stocks are in bold. The unique monophyletic lineage corresponds to *Trypanosoma brucei gambiense* and is indicated above the corresponding branch. The presence of several missing genotypes prohibited the use of bootstraps. Bo = Bonon, Si = Sinfra, Tbg = *Trypanosoma brucei gambiense*, Tbg2 = *Trypanosoma brucei gambiense* group 2, Tbb = *Trypanosoma brucei brucei*, Tbrh = *Trypanosoma brucei rhodesiense*.

Among the 76 DNA samples positive in the TBR PCR, only 10 showed amplification in the microsatellite genotyping assays. The NJTree presented in Fig 3 presents a classical shape (e.g. [42])) with one monophyletic lineage that gathers all *T*. *b*. *gambiense* reference stocks and rake for other subspecies. No trypanosome genotypes from the domestic animals sampled in Bonon and Sinfra foci are members of this *T*. *b*. *gambiense* lineage.

### Trypanolysis

**Fig 4:**
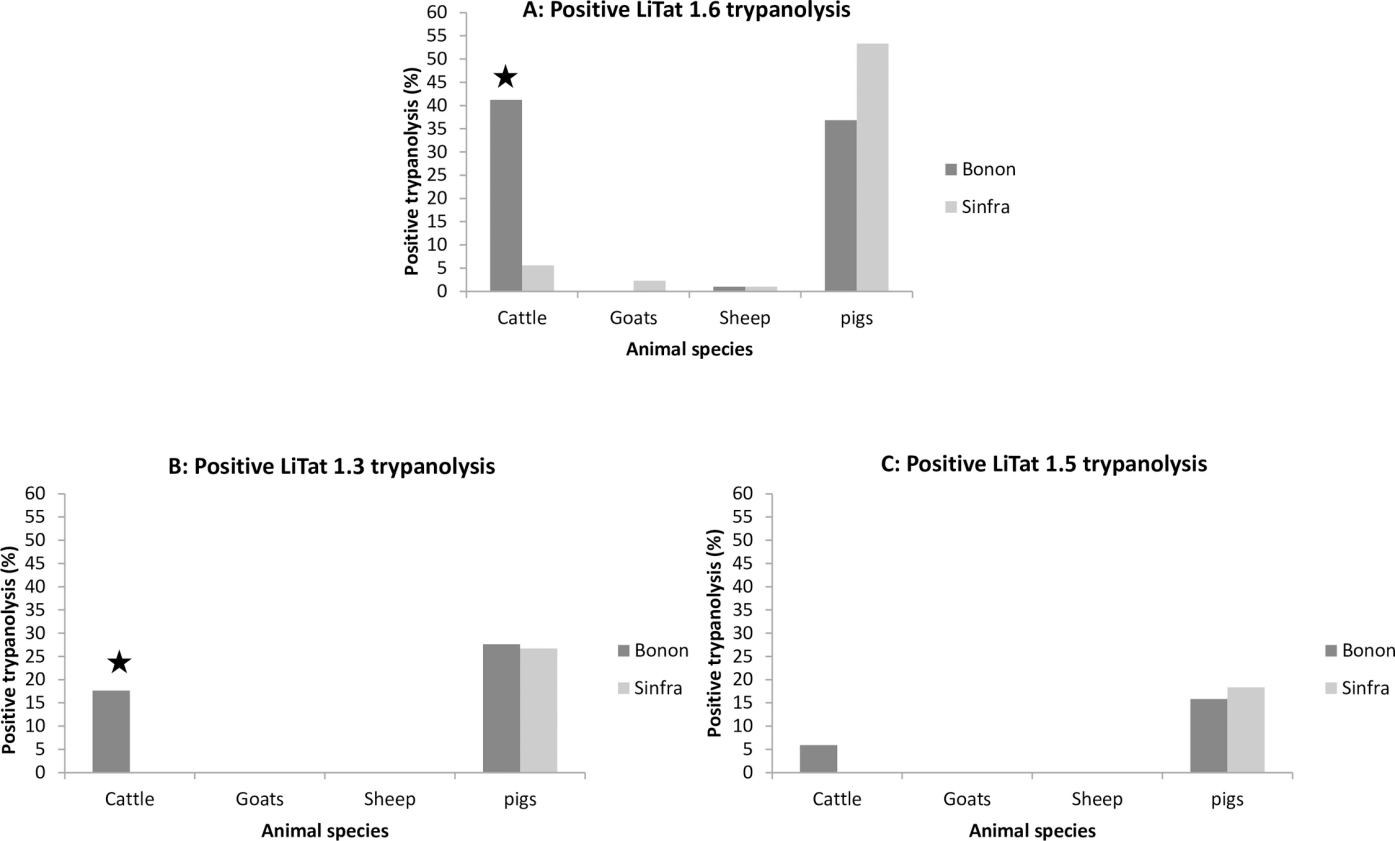
Immune trypanolysis (TL) results. Proportion of the LiTat 1.6 (4A), LiTat 1.3 (4B) and LiTat 1.5 (4C) TL positive results on the total sample collection for each host in the two foci. A significant difference between Bonon and Sinfra is indicated with a star.

Trypanolysis results for the three VAT types per host in the two foci are presented in Fig 4. No TL positive results were observed in goats and sheep with LiTat 1.3 and 1.5 VAT in both foci and only two goats and two sheep were positive with the LiTat 1.6 VAT. The LiTat 1.6 TL assay showed high positivity rates (more than 35%) in pigs in both foci and in cattle (more than 40%) in the Bonon focus, confirming the PCR-TBR results. We observed the same pattern, but with lower positivity rates, in the *T*. *b*. *gambiense*-specific LiTat 1.3 and 1.5 TL assays (0% for both 1.3 and 1.5 in cattle in Sinfra, 17.6 and 5.9% for 1.3 and 1.5 respectively in cattle in Bonon and between 15.8 and 27.6% for both 1.3 and 1.5 in pigs in the two foci). The difference observed in cattle between Bonon and Sinfra is significant for Litat 1.3.

**Fig 5:**
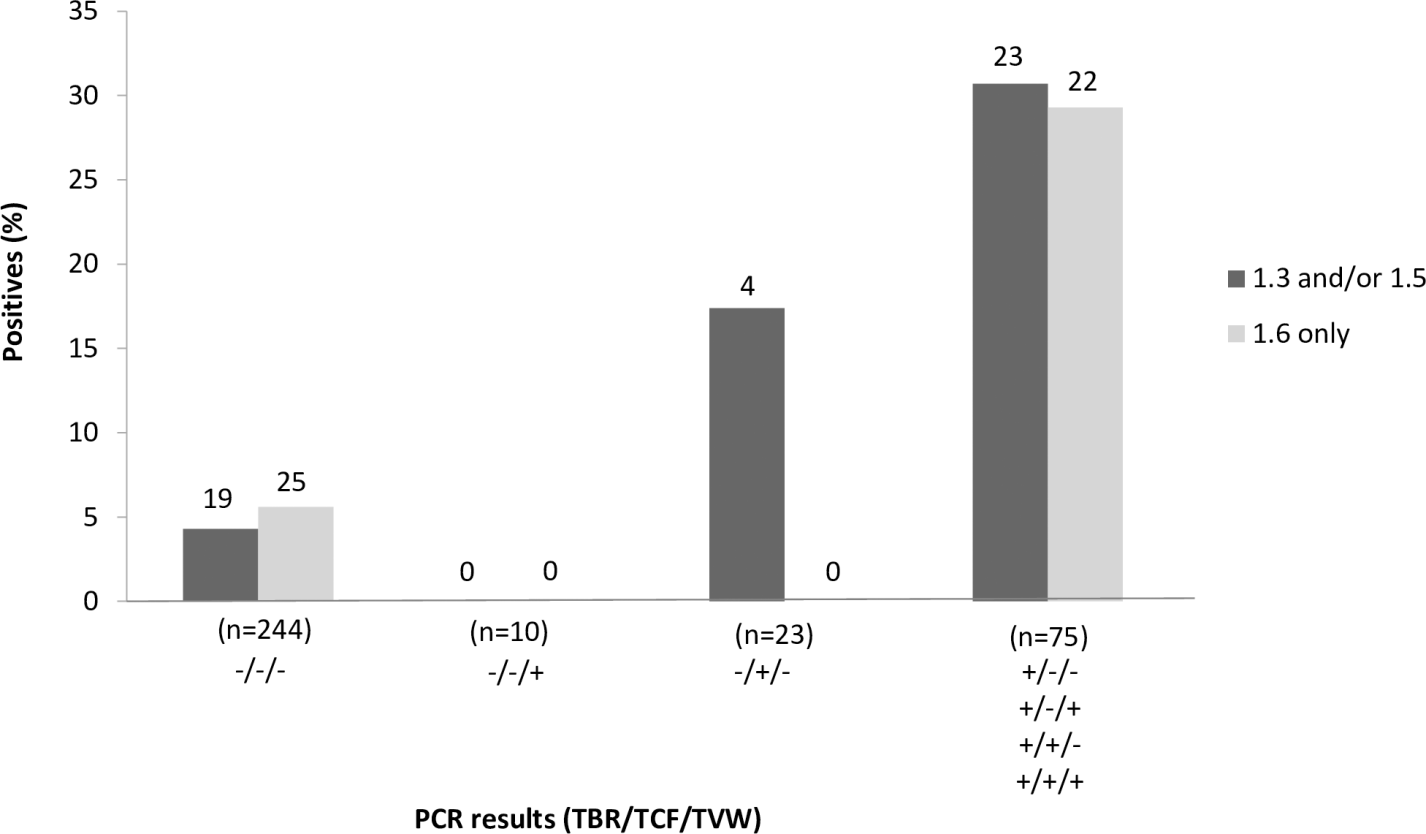
Reactivity to the Litat 1.3, 1.5 and 1.6 VAT according to the different PCR profiles. PCR profiles are given as follow: PCR TBR result/PCR TCF result/PCR TVW result. n = number of animals with the corresponding profile. Numbers on the top are the numbers of animal positives with Litat 1.3 and/or 1.5 TL or with LiTat 1.6 only.

The distribution of LiTat 1.3 and/or LiTat 1.5 TL positive results in cattle (7 individuals = 8%) and pigs (39 individuals = 28.5%) is given in Table 3. Out of the seven cattle which tested positive to either LiTat 1.3 or LiTat 1.5, only one was positive to both variants (13.3%). This proportion was much higher in pigs with 21 of 39 (53.9%) positive animals tested positive to both variants. From these 21 pigs, 15 (10.9%) were also positive to LiTat 1.6 and thus positive to the three VAT types. Animals testing positive to LiTat 1.6 only were also observed for all domestic animal species but with higher proportion in cattle (13.8%) and pigs (22.6%).Nineteen of the 46 samples (43.3%) testing positive to Litat1.3 and/or 1.5 were negative in all species specific PCR assays (TBR, TCF and TVW). Positivity to Litat1.3 and/or 1.5 was the highest in animals testing positive to the TBR PCR (30.6%), it was zero in animals positive only to TVW PCR, but positive in 4/23 (17.4%) animals that were uniquely positive to TCF PCR (Fig 5).

**Table 3:** Distribution of the LiTat 1.3 and/or LiTat 1.5 trypanolysis positive results in cattle and pigs.

		TL profiles (LiTat 1.3/LiTat1.5/LiTat1.6)					
Animal species		(-/+/+)	(+/-/-)	(+/-/+)	(+/+/-)	(+/+/+)	Total
Cattle	n	1	2	3	0	1	7
	% positive	1.15	2.30	3.45	0	1.15	8.05
Pigs	n	2	4	12	6	15	39
	% positive	1.46	2.92	8.76	4.38	10.95	28.47

## Discussion

In this study, we showed that domestic animals are important carriers of trypanosomes in the Sinfra and Bonon HAT foci. *T*. *brucei* s.l. infections were highest in pigs in Sinfra and both in pigs and cattle in Bonon. The overall prevalence recorded by PCR was approximately twice the one observed with the parasitological BCT technique. This was expected due to the known higher sensitivity of PCR [28,43]. Infection rates were higher in pigs and cattle than in sheep and goats. This may be related to differences in the host-vector contacts associated with different breeding practices and/or to differential host susceptibility to trypanosome infection. Sheep rather graze in the vicinity of the houses where they are partly nourished by the villagers, limiting the contact with tsetse flies. Goats are browsing freely across the vegetation in the periphery of villages and may be more exposed to tsetse flies. Noteworthy, low trypanosome infection rates have already been described for goats and this was attributed partly to the fact that goats are known to express individual defensive behavior against the bites of tsetse flies [43,44]. Cattle forage across long distances to reach pastures and are thus potentially more exposed to tsetse flies. However, in the Sinfra focus, the areas crossed by cattle herds are particularly anthropized with lower tsetse fly infestations. In contrast, in Bonon, the forest was more recently exploited and cattle are still in contact with tsetse populations [10]. This probably explains why cattle infection rates in Bonon are much higher than in Sinfra. Pigs roam freely in the more humid and shady areas around the villages or along the small rivers and are highly exposed to tsetse flies. Moreover, pigs have already been described as a preferential feeding host for *Glossina palpalis palpalis* [26,45], the only tsetse species present in the studied areas [26].

In the Sinfra and Bonon areas, the forest was progressively replaced by cocoa, coffee, banana plantations and food crops offering potential favorable conditions to the introduction of *T*. *congolense* savannah type from the north of the country where prevalence of this species is high [46]. However we did not detect this trypanosome which is considered as the most pathogenic *congolense* type [47], in the framework of this study. The prevalence of *T*. *vivax* was also low with only 13 infections, mainly detected in sheep. Noteworthy, no *T*. *vivax* infections were detected in pigs, confirming that this host is generally refractory to this trypanosome species [48]. *T*. *congolense* forest type was found in all host animals as previously observed in other forest areas [49] and mainly detected in pigs. *T*. *brucei* s.l. was the predominant species detected in our study areas, which is in line with studies conducted in other HAT forest foci from Cameroon and Equatorial Guinea [17,50].

Among the *T*. *brucei* s.l. infections, *T*. *b*. *gambiense* could not be detected by the TgsGP PCR nor by microsatellite genotyping. At first sight, this could indicate that the human pathogenic trypanosome is not circulating in domestic animals in the HAT foci of Bonon and Sinfra. Similar results were reported in northwest Uganda where authors concluded an apparent absence of domestic animal reservoir for *T*. *b*. *gambiense* [51]. However, we observed high positivity rates (up to 28%) with both the *T*. *b*. *gambiense* specific LiTat 1.3 and LiTat 1.5 TL assays. Positivity to more than one *T*. *b*. *gambiense* variant is an indicator that the host was infected for a sufficient amount of time to allow VSG switching. Together, the high prevalence of LiTat 1.3 and/or 1.5 positivity observed in pigs and the fact that more than half of the animal tested positive to both variants, are thus pointing out that pigs could be potential reservoirs of *T*. *b*. *gambiense* in the study area.

Since very few HAT cases have been identified in these foci in the last years (Fig 1), the TL results may suggest an active circulation of *T*. *b*. *gambiense* in pigs and/or cattle in the Sinfra and Bonon foci, but with little contact with humans. In most studies describing the presence of *T*. *b*. *gambiense* in wild and domestic animals, prevalence in animals is often higher than in humans [18,19,50,52]. This may be explained by a higher nutritional preference of tsetse flies for animals and by the fact that human are less exposed to tsetse flies in these areas. This is consistent with previous observations describing the protective role of pigs living at the periphery of villages since pigs are the preferential host of tsetse flies [24–26,53].

The TL results obtained in our study are consistent with recent population genetics data which showed that natural populations of *T*. *b*. *gambiense* are more important than those evidenced by the classical medical survey, suggesting hidden reservoirs of these parasites [42,54]. In the same way, a recent modeling study with animal and human data from a Cameroon focus showed that transmission of *T*. *b*. *gambiense* could not be maintained by humans as the only reservoir [55]. The existence of a domestic animal reservoir for *T*. *b*. *gambiense* could be responsible for the sporadic cases diagnosed in most of the Ivorian forest foci, sometimes a long time after the last epidemic HAT episode [7].

We observed a high discordance between the *T*. *b*. *gambiense* PCR and TL results. The *T*. *b*. *gambiense* specific TgsGP PCR targets a single copy gene [56] and may not be sensitive enough to exclude presence of *T*. *b*. *gambiense* in case of negative result, given the low parasitaemia generally observed in *T*. *b*. *gambiense* infections [57,58]. In addition, the microsatellite genotyping assays are known to lack sensitivity [59]. It is thus possible that *T*. *b*. *gambiense* parasites could have been missed by these two methods. In addition, the skin could be an important anatomical reservoir of *T*. *b*. *gambiense* as was recently demonstrated in experimentally infected mice [60–62]. We can thus not exclude that animals with negative PCR results on blood have trypanosomes in other body compartments such as the dermis. It is also possible that the presence of LiTat 1.3 and/or LiTat 1.5 specific antibodies indicates a previous transient infection. In addition, cross reactions of Litat 1.3 and 1.5 with other trypanosome species cannot be excluded. Bromidge et al. showed in 1993 that a PCR targeting the LiTat 1.3 gene showed a positive result in two stocks described as *T*. *brucei brucei* isolated from pigs in Côte d’Ivoire [63]. We thus cannot exclude that *T*. *b*. *brucei* strains circulating in our study areas are expressing the LiTat 1.3 VAT resulting in TL positive results.

In the context of HAT elimination, it will be crucial to improve the sensitivity of the *T*. *b*. *gambiense* PCR in order to detect this species in biological samples of animals, tsetse flies and human. Further studies are also needed to validate the specificity of LiTat 1.3 and 1.5 TL assays in animals. Controlled infection experiments in domestic animals will be needed to more fully evaluate the specificity and sensitivity of the currently available tools and evaluate their usefulness in research on the role of animals in the transmission of *T*. *b*. *gambiense*.

Our data suggest the existence of a potential domestic animal reservoir for *T*. *b*. *gambiense* HAT and provides indications for areas where the transmission may occur in the Sinfra and Bonon foci: the small wet and shady areas around the villages and the forest relics. Vector control using tiny targets [64,65] which are particularly favorable to disrupt the contact between domestic animals and tsetse flies may have a considerable impact on tsetse fly densities and trypanosome transmission in these areas. The concomitant treatment of pigs in the Sinfra focus and both pigs and cattle in the Bonon focus would furthermore help clearing out the potential reservoir of *T*. *b*. *gambiense* but also would contribute to animal trypanosomiasis control. This clearly illustrates the usefulness of applying a one health strategy for both HAT and AAT control.

However, these control measures have to be adapted to the study area and epidemiological context. In the endemic focus of Boffa [66] and in the historical focus of Loos Islands [67] in Guinea, domestic animals seem not to play a role in the epidemiology of HAT. In Cameroun [49,50,68], Congo [69], and Equatorial Guinea [17–19], the presence of human and animal infecting trypanosomes in domestic and/or wild animals could be evidenced, but important differences between study areas were highlighted regarding prevalence in hosts and their geographical distribution. We thus suggest, in low prevalence foci where HAT elimination seems reachable, to conduct animal surveys to define the most appropriate control measures to be implemented.

## Conclusion

We have investigated the distribution of animal trypanosomes and the possible existence of a domestic animal reservoir of *T*. *b*. *gambiense* in two hypo-endemic HAT foci in Côte d’Ivoire. Our results show that *T*. *brucei* s.l. and *T*. *congolense* forest type circulate in the study areas and mainly infect pigs and cattle. Discordant results were obtained on the presence of *T*. *b*. *gambiense*, between PCR and TL methods. PCR did not detect *T*. *b*. *gambiense* while high seroprevalence was observed in TL. In the context of HAT elimination, it will be crucial to further investigate this discordance and to develop better tools and strategies to fully characterize the epidemiological role of an animal reservoir for *T*. *b*. *gambiense*.
